# ExoNet Database: Wearable Camera Images of Human Locomotion Environments

**DOI:** 10.3389/frobt.2020.562061

**Published:** 2020-12-03

**Authors:** Brock Laschowski, William McNally, Alexander Wong, John McPhee

**Affiliations:** ^1^Department of Systems Design Engineering, University of Waterloo, Waterloo, ON, Canada; ^2^Waterloo Artificial Intelligence Institute, University of Waterloo, Waterloo, ON, Canada

**Keywords:** artificial intelligence, environment recognition, exoskeletons, rehabilitation robotics, prosthetics, biomechatronics, computer vision, wearable technology

## Introduction

Hundreds of millions of individuals worldwide have mobility impairments resulting from degenerative aging and/or neuro-musculoskeletal disorders (Grimmer et al., 2019). Fortunately, robotic lower-limb exoskeletons and prostheses can allow otherwise wheelchair-bound seniors and rehabilitation patients to perform movements that involve net positive mechanical work (e.g., climbing stairs and standing from a seated position) using onboard actuators and intelligent control systems (Tucker et al., 2015; Young and Ferris, 2017; Laschowski and Andrysek, 2018; Krausz and Hargrove, 2019; Zhang et al., 2019a). Generally speaking, the high-level controller recognizes the patient's locomotion mode (intention) by analyzing real-time measurements from wearable sensors using machine learning algorithms. The mid-level controller then translates the locomotion intentions into mode-specific reference trajectories. This control level typically comprises a finite state machine, which implements a discrete parametrized control law (e.g., joint position or mechanical impedance control) for each locomotion mode. Finally, the low-level controller tracks the reference trajectories and minimizes the signal error by modulating the device actuators using feedforward and feedback control loops (Tucker et al., 2015; Young and Ferris, 2017; Laschowski and Andrysek, 2018; Krausz and Hargrove, 2019; Zhang et al., 2019a).

Accurate transitions between different locomotion modes is important since even rare misclassifications can cause loss-of-balance and injury. In many commercial devices like the ReWalk and Indego lower-limb exoskeletons, the patient acts as the high-level controller by performing volitional movements to manually switch between locomotion modes (Tucker et al., 2015; Young and Ferris, 2017). These human-controlled methods can be time-consuming, inconvenient, and cognitively demanding. Researchers have recently developed automated locomotion mode recognition systems using wearable sensors like inertial measurement units (IMUs) and surface electromyography (EMG) to automatically switch between different locomotion modes (Tucker et al., 2015; Young and Ferris, 2017; Laschowski and Andrysek, 2018; Krausz and Hargrove, 2019; Zhang et al., 2019a). Whereas mechanical and inertial sensors respond to the patient's movements, the electrical potentials of biological muscles, as recorded using surface EMG, precede movement initiation and thus could (marginally) predict locomotion mode transitions. Several researchers have combined mechanical sensors with surface EMG for automated locomotion mode recognition. Such neuromuscular-mechanical data fusion has improved the locomotion mode recognition accuracies and decision times compared to implementing either system individually (Huang et al., 2011; Du et al., 2012; Wang et al., 2013; Liu et al., 2016). However, these measurements are still patient-dependent, and surface EMG are susceptible to fatigue, changes in electrode-skin conductivity, and crosstalk from adjacent muscles (Tucker et al., 2015).

Supplementing neuromuscular-mechanical data with information about the upcoming walking environment could improve the high-level control performance. Similar to the human visual system, environment sensing would precede modulation of the patient's muscle activations and/or walking biomechanics, therein enabling more accurate and real-time locomotion mode transitions. Environment sensing could also be used to adapt low-level reference trajectories (e.g., changing toe clearance corresponding to an obstacle height) (Zhang et al., 2020) and optimal path planning (e.g., identifying opportunities for energy regeneration) (Laschowski et al., 2019a, 2020a). Preliminary research has shown that supplementing an automated locomotion mode recognition system with environment information can improve the classification accuracies and decision times compared to excluding terrain information (Huang et al., 2011; Wang et al., 2013; Liu et al., 2016). Several researchers have explored using radar detectors (Kleiner et al., 2018) and laser rangefinders (Zhang et al., 2011; Wang et al., 2013; Liu et al., 2016) for environment sensing. However, vision-based systems can provide more detailed information about the field-of-view and detect physical obstacles in peripheral locations. Most environment recognition systems have included either RGB cameras (Krausz and Hargrove, 2015; Diaz et al., 2018; Khademi and Simon, 2019; Laschowski et al., 2019b; Novo-Torres et al., 2019; Da Silva et al., 2020; Zhong et al., 2020) or 3D depth cameras (Krausz et al., 2015, 2019; Varol and Massalin, 2016; Hu et al., 2018; Massalin et al., 2018; Zhang et al., 2019b,c,d).

For image classification, researchers have used learning-based algorithms like support vector machines (Varol and Massalin, 2016; Massalin et al., 2018) and deep convolutional neural networks (Rai and Rombokas, 2018; Khademi and Simon, 2019; Laschowski et al., 2019b; Novo-Torres et al., 2019; Zhang et al., 2019b,c,d; Zhong et al., 2020). Although convolutional neural networks typically outperform support vector machines for image classification (LeCun et al., 2015), deep learning requires significant and diverse training images to prevent overfitting and promote generalization. Deep learning has become pervasive ever since AlexNet (Krizhevsky et al., 2012) popularized convolutional neural networks by winning the 2012 ImageNet challenge. ImageNet is an open-source dataset containing ~15 million labeled images and 22,000 different classes (Deng et al., 2009). The lack of an open-source, large-scale dataset of human locomotion environment images has impeded the development of environment-aware control systems for robotic lower-limb exoskeletons and prostheses. Until now, researchers have been required to individually collect training images to develop their classification algorithms. These repetitive measurements are time-consuming and inefficient, and individual private datasets have prevented comparisons between classification algorithms from different researchers (Laschowski et al., 2020b). Drawing inspiration from ImageNet, we developed ExoNet–the first open-source, large-scale hierarchical database of high-resolution wearable camera images of human walking environments. In accordance with the Frontiers submission guidelines, this article provides a detailed description of the research dataset. Benchmark performance and analyses of the ExoNet database for human locomotion environment classification will be presented in future work.

## Materials and Methods

### Large-Scale Data Collection

**Figure 1:**
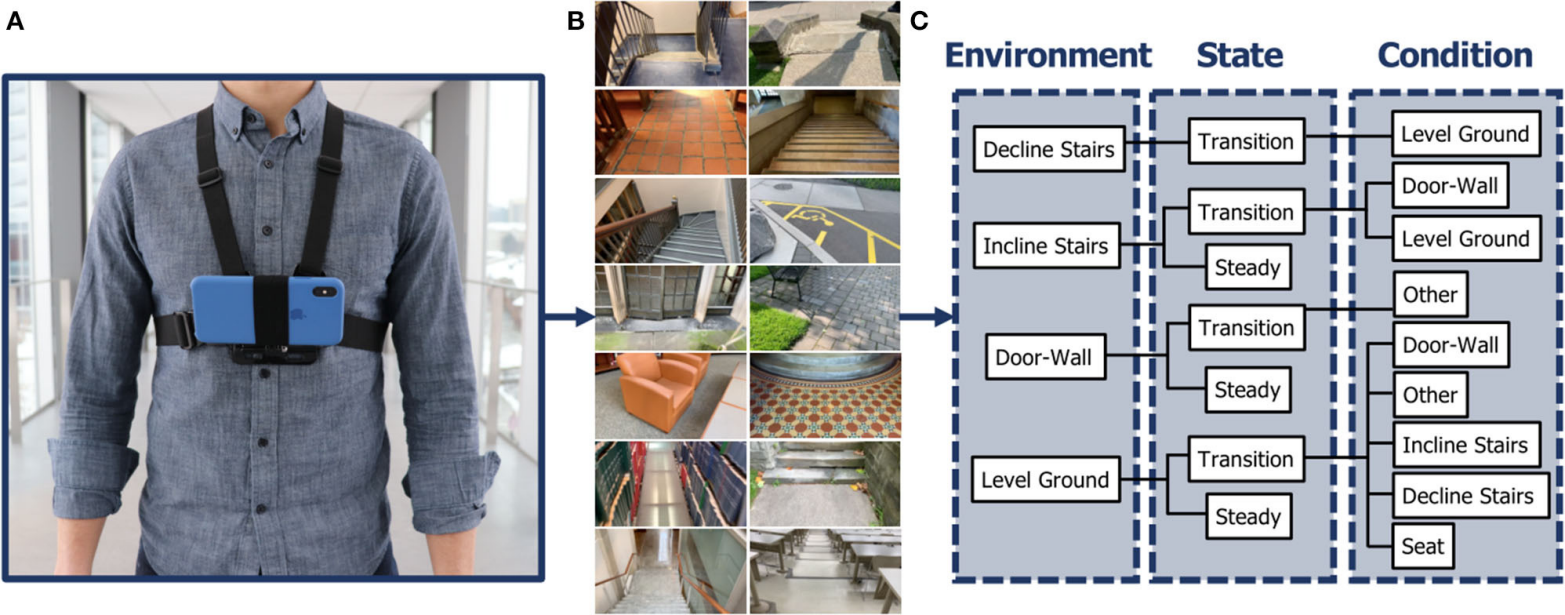
Development of the ExoNet database, including **(A)** photograph of the wearable camera system used for large-scale data collection; **(B)** examples of the high-resolution RGB images (1,280 × 720) of human walking environments; and **(C)** schematic of the 12-class hierarchical labeling architecture.

One subject was instrumented with a lightweight wearable smartphone camera system (iPhone XS Max); photograph shown in Figure 1A. Unlike limb-mounted systems (Zhang et al., 2011, 2019b,c; Varol and Massalin, 2016; Diaz et al., 2018; Hu et al., 2018; Kleiner et al., 2018; Massalin et al., 2018; Rai and Rombokas, 2018; Da Silva et al., 2020), chest-mounting can provide more stable video recording and allow users to wear pants and long dresses without obstructing the sampled field-of-view. The chest-mount height was ~1.3 m from the ground when the participant stood upright. The smartphone contains two 12-megapixel RGB rear-facing cameras and one 7-megapixel front-facing camera. The front and rear cameras provide 1,920 × 1,080 and 1,280 × 720 video recording at 30 frames/s, respectively. The smartphone weighs ~0.21 kg, and features an onboard rechargeable lithium-ion battery, 512-GB of memory storage, and a 64-bit ARM-based integrated circuit (Apple A12 Bionic) with six-core CPU and four-core GPU. These hardware specifications can support onboard machine learning for real-time environment classification. The relatively lightweight and unobtrusive nature of the wearable camera system allowed for unimpeded human walking biomechanics. Ethical review and approval were not required for this research in accordance with the University of Waterloo Office of Research Ethics.

While most environment recognition systems have been limited to controlled indoor environments and/or prearranged walking circuits (Zhang et al., 2011, 2019b,c,d; Du et al., 2012; Wang et al., 2013; Krausz et al., 2015, 2019; Liu et al., 2016; Hu et al., 2018; Kleiner et al., 2018; Khademi and Simon, 2019), our subject walked around unknown outdoor and indoor real-world environments while collecting images with occlusions, signal noise, and intraclass variations. Data were collected at various times throughout the day to incorporate different lighting conditions. Similar to human gaze fixation during walking (Li et al., 2019), the sampled field-of-view was ~1–5 meters ahead of the participant, thereby showing upcoming walking environments rather than the ground underneath the subject's feet. The camera's pitch angle slightly differed between data collection sessions. Images were sampled at 30 Hz with 1,280 × 720 resolution. More than 52 h of video were recorded, amounting to ~5.6 million images (examples shown in Figure 1B). The same environment was never sampled twice to maximize diversity among the ExoNet images. Data were collected throughout the summer, fall, and winter seasons to incorporate different weathered surfaces like snow, grass, and multicolored leaves. In accordance with the Frontiers submission guidelines, the ExoNet database was deposited in a public repository (IEEE DataPort) and is available for download at https://ieee-dataport.org/open-access/exonet-database-wearable-camera-images-human-locomotion-environments. The file size of the uncompressed videos is ~140 GB.

### Hierarchical Image Labeling

Given the subject's preferred walking speed, there were minimal differences between consecutive images sampled at 30 Hz. The labeled images were therefore downsampled to 5 frames/s to minimize the demands of manual annotation and increase the diversity in image appearances. However, for real-time environment classification and control of robotic lower-limb exoskeletons and prostheses, higher sampling rates would be more advantageous for accurate locomotion mode recognition and transitioning. Similar to ImageNet (Deng et al., 2009), the ExoNet database was human-annotated using a hierarchical labeling architecture (see Figure 1C). Images were labeled according to exoskeleton and prosthesis control functionality, rather than a purely computer vision perspective. For instance, images of level-ground environments showing either pavement or grass were not differentiated since both surfaces would use the same level-ground walking state controller. In contrast, computer vision researchers might label these different surface textures as separate classes.

Approximately 923,000 images in ExoNet were manually labeled and organized into 12 classes using the following descriptions, which also include the number of labeled images/class: {IS-T-DW = 31,628} shows incline stairs with a door and/or wall; {IS-T-LG = 11,040} shows incline stairs with level-ground thereafter; {IS-S = 17,358} shows only incline stairs; {DS-T-LG = 28,677} shows decline stairs with level-ground thereafter; {DW-T-O = 19,150} shows a door and/or wall with *other* (e.g., hand or window); {DW-S = 36,710} shows only a door and/or wall; {LG-T-DW = 379,199} shows level-ground with a door and/or wall; {LG-T-O = 153,263} shows level-ground with *other* (e.g., humans, cars, bicycles, or garbage cans); {LG-T-IS = 26,067} shows level-ground with incline stairs thereafter; {LG-T-DS = 22,607} shows level-ground with decline stairs thereafter; {LG-T-SE = 119,515} shows level-ground with seats (e.g., couches, chairs, or benches); and {LG-S = 77,576} shows only level-ground. These classes were selected to encompass the different walking environments encountered during the data collection sessions. We included the *other* class to improve image classification performance when confronted with non-terrain related features like humans or bicycles.

Inspired by previous work (Huang et al., 2011; Du et al., 2012; Wang et al., 2013; Liu et al., 2016; Khademi and Simon, 2019), the hierarchical labeling architecture included both *steady* (S) and *transition* (T) states. A steady state describes an environment where an exoskeleton or prosthesis user would continuously perform the same locomotion mode (e.g., only level-ground terrain). In contrast, a transition state describes an environment where the exoskeleton or prosthesis high-level controller might switch between different locomotion modes (e.g., level-ground and incline stairs). Manually labeling the transition states was relatively subjective. For example, an image showing level-ground terrain was labeled *level-ground-transition-incline stairs* (LG-T-IS) when an incline staircase was approximately within the sampled field-of-view and forward-facing. Similar labeling principles were applied to transitions to other conditions. The Python code used for labeling the ExoNet database was uploaded to GitHub and is publicly available for download at https://github.com/BrockLaschowski2/ExoNet.

## Discussion

Environment recognition systems can improve the control of robotic lower-limb exoskeletons and prostheses during human locomotion. However, small-scale and private training datasets have impeded the widespread development and dissemination of image classification algorithms for human locomotion environment recognition. Motivated by these limitations, we developed ExoNet–the first open-source, large-scale hierarchical database of high-resolution wearable camera images of human walking environments. Using a lightweight wearable camera system, we collected over 5.6 million RGB images of different indoor and outdoor real-world walking environments, of which ~923,000 images were human-annotated using a 12-class hierarchical labeling architecture. Available publicly through IEEE DataPort, ExoNet provides researchers an unprecedented communal platform to develop and compare next-generation image classification algorithms for human locomotion environment recognition. Although ExoNet was originally designed for environment-aware control systems for lower-limb exoskeletons and prostheses, applications could extend to humanoids and autonomous legged robots (Park et al., 2015; Villarreal et al., 2020). Users of the ExoNet database are requested to reference this dataset report.

Aside from being the only open-source image database of human locomotion environments, the large scale and diversity of ExoNet significantly distinguishes itself from previous environment recognition systems, as illustrated in Table 1. ExoNet contains ~923,000 individually labeled images. In comparison, the previous largest dataset contained ~402,000 images (Massalin et al., 2018). While most environment recognition systems have included fewer than six classes (Krausz and Hargrove, 2015; Krausz et al., 2015, 2019; Varol and Massalin, 2016; Massalin et al., 2018; Khademi and Simon, 2019; Laschowski et al., 2019b; Novo-Torres et al., 2019; Zhang et al., 2019b,c,d; Zhang et al., 2020), the ExoNet database features a 12-class hierarchical labeling architecture. These differences have real-world implications given that learning-based algorithms like convolutional neural networks require significant and diverse training images (LeCun et al., 2015). The spatial resolution of the ExoNet images (1,280 × 720) is considerably higher than previous efforts (e.g., 224 × 224 and 320 × 240). Poor image resolution has been attributed to decreased classification accuracy of human walking environments (Novo-Torres et al., 2019). Although higher resolution images can increase the computational and memory storage requirements, that being unfavorable for real-time mobile computing, researchers have been moving toward the development of efficient convolutional neural networks that require fewer operations (Tan and Le, 2020), therein enabling the processing of larger images for relatively similar computational power. Here we assume mobile computing for the exoskeleton and prosthesis control (i.e., untethered and no wireless communication to cloud computing). Nevertheless, an exoskeleton or prosthesis controller may not always benefit from additional information provided by higher resolution images, particularly when interacting with single surface textures (i.e., only pavement or grass). With ongoing research and development in computer vision and artificial intelligence, larger and more challenging training datasets are needed to develop better image classification algorithms for environment-aware locomotor control systems.

**Table 1:** Comparison of the ExoNet database with previous environment recognition systems for robotic lower-limb prostheses and exoskeletons.

**Reference**	**Sensor**	**Position**	**Dataset**	**Resolution**	**Classes**
Da Silva et al. (2020)	RGB camera	Lower-limb	3,992 Images	512 × 512	6
Diaz et al. (2018)	RGB camera	Lower-limb	3,992 Images	1,080 × 1,920	6
Khademi and Simon (2019)	RGB camera	Waist	7,284 Images	224 × 224	3
Krausz and Hargrove (2015)	RGB camera	Head	5 Images	928 × 620	2
Krausz et al. (2015)	Depth camera	Chest	170 Images	80 × 60	2
Krausz et al. (2019)	Depth camera	Waist	4,000 Images	171 × 224	5
Laschowski et al. (2019b)	RGB camera	Chest	34,254 Images	224 × 224	3
Massalin et al. (2018)	Depth camera	Lower-limb	402,403 Images	320 × 240	5
Novo-Torres et al. (2019)	RGB camera	Head	40,743 Images	128 × 128	2
Varol and Massalin (2016)	Depth camera	Lower-limb	22,932 Images	320 × 240	5
Zhang et al. (2019b,c)	Depth camera	Lower-limb	7,500 Images	224 × 171	5
Zhang et al. (2019d)	Depth camera	Waist	4,016 Images	2,048 Point Cloud	3
Zhang et al. (2020)	Depth camera	Lower-limb	7,500 Images	100 × 100	5
Zhong et al. (2020)	RGB camera	Head and lower-limb	327,000 Images	1,240 × 1,080	6
**ExoNet database**	**RGB camera**	**Chest**	**922,790 Images**	**1,280** **×** **720**	**12**

A potential limitation of the ExoNet database is the two-dimensional nature of the environment information. Whereas RGB cameras measure light intensity information, depth cameras also provide distance measurements (Krausz et al., 2015, 2019; Varol and Massalin, 2016; Hu et al., 2018; Massalin et al., 2018; Zhang et al., 2019b,c,d). Depth cameras work by emitting infrared light and calculate distances by measuring the light time-of-flight between the camera and physical environment (Varol and Massalin, 2016). Depth measurement accuracies typically degrade in outdoor lighting conditions (e.g., sunlight) and with increasing measurement distance. Consequently, most environment recognition systems using depth cameras have been tested in indoor environments (Krausz et al., 2015, 2019; Varol and Massalin, 2016; Hu et al., 2018; Massalin et al., 2018) and have had limited capture volumes (i.e., between 1 and 2 m of maximum range imaging) (Krausz et al., 2015; Varol and Massalin, 2016; Massalin et al., 2018). Assuming mobile computing, the application of depth cameras for environment sensing would also require robotic lower-limb exoskeletons and prostheses to have embedded microcontrollers with significant computing power and minimal power consumption, the specifications of which are not supported by existing untethered systems (Massalin et al., 2018). These practical limitations motivated our decision to use RGB images.

Our camera images could be fused with the smartphone IMU measurements to improve high-level control performance. For example, if an exoskeleton or prosthesis user unexpectedly stops while walking toward an incline staircase, the acceleration measurements would indicate static standing rather than stair ascent, despite the staircase being accurately detected in the field-of-view. Since environment information does not explicitly represent the locomotor intent, environment recognition systems should supplement, rather than replace, the automated locomotion mode recognition systems based on patient-dependant measurements like mechanical and inertial sensors. The smartphone IMU measurements could also be used for sampling rate control (Zhang et al., 2011; Diaz et al., 2018; Khademi and Simon, 2019; Da Silva et al., 2020). Faster walking speeds would likely benefit from higher sampling rates for continuous classification. In contrast, static standing does not necessarily require environment information and therefore the smartphone camera could be powered down, or the sampling rate decreased, to minimize the computational and memory storage requirements. However, the optimal method for fusing the smartphone camera images with the onboard IMU measurements remains to be determined.

## Data Availability Statement

The datasets presented in this study can be found in online repositories. The names of the repository/repositories and accession number(s) can be found below: https://ieee-dataport.org/open-access/exonet-database-wearable-camera-images-human-locomotion-environments. The name of the online repository is “IEEE DataPort” and the name of the image database is “ExoNet”.

## Ethics Statement

Ethical review and approval was not required for the study on human participants in accordance with the local legislation and institutional requirements. Written informed consent for participation was not required for this study in accordance with the national legislation and the institutional requirements.

## Author Contributions

BL was responsible for the study design, literature review, data collection, image labeling, data interpretation, and manuscript writing. WM assisted with the study design, image labeling, data interpretation, and manuscript writing. AW and JM assisted with the study design, data interpretation, and manuscript writing. All authors read and approved the final manuscript.

## Conflict of Interest

The authors declare that the research was conducted in the absence of any commercial or financial relationships that could be construed as a potential conflict of interest.
